# Supplementary material for: Laboratory-confirmed influenza infection and acute myocardial infarction among United States senior Veterans
Source: PLoS One. 2020 Dec 11;15(12):e0243248. doi: 10.1371/journal.pone.0243248 (PMC7732109; doi:10.1371/journal.pone.0243248)
Supplement: Attachment — Submitted filename: Response to reviewers comments rev1 10-13-20 yyx.docx [file pone.0243248.s001.docx]

**Reviewer #1**: It is a well-written article, although it was adapted from a previous study with similar design. It is somewhat lacking in originality and appealing new findings. I believe further analysis and edits and could improve the quality of this paper.

Thank you very much for your encouragement and kind words. Your suggestions and questions are well taken, and we worked hard to improve the quality of our paper. Although part of this article is about our effort at replicating others’ findings for the first time in the United States, and in an unique population – senior Veterans, we also are the first to examine and quantify, instead of hypothesizing, the underlying biomechanism with laboratory data which could pave the way for pharmaceutical interventions. We believe the seeming lack of originality and new findings is because we did a poor job at presenting our findings in the appropriate context. Along with addressing your specific comments, we have invested much effort in remedying this appearance.

1. In line number 135-137 and 143, the authors described Laboratory confirmed influenza as the exposure variable and acute MI hospitalization as the outcome variable for the purpose of this study. The authors concluded that they found “significant association between LCI and AMI among senior Veterans”. However, they have compared with the “control interval” which is 52 weeks before the index date (Influenza test date) and 51 weeks after the end of the risk interval (the first 7 days after the index date). In general, exposure should be prior to any outcome for measurement of an association between exposure and outcome. How AMI episode before the influenza test can be included for association analysis? I think there is proper explanation and authors could consider including this clearly in the methods section.

That is an excellent question. Sorry we were not clear. An AMI episode that occurred before an influenza test is exactly the scenario that was included in Figure 1 (Veteran B). These events contribute to the estimation of a reference baseline AMI rate, in other words, event rates during the control period. In a self-controlled case series design, the method of examining association is self-comparison: a subject’s event rate during the risk period, in this case one week after lab confirmed influenza infection, is compared to the same subject’s event rate during the control period. One thing that was not clearly explained was that, during the control period, there could not be AMI within 7 days after an influenza infection, otherwise that would be counted in the risk period. Consequently, the exposure (influenza infection) is always before the AMI during the risk period. If there are other exposures before the AMI, although they are not specified, e.g., snow removal, they would have been included in the control period. We now have included this explanation right below the first mention of Figure 1, in the paragraph titled Statistical Analysis. (page 6, lines 161-168)

2. More than half of the patients had known high risk factors for CVD (table 1). It would be good to see if patients with the similar co morbidity were stratified and compared for AMI after LCI.

Agreed. We conducted additional stratified analysis for the following variables: Dyslipidemia, Diabetes, and Hypertension (table 2). These results were presented in the Results section (starting on page 11, lines 270-281) and discussed in the Discussion section (page 16, lines 382-385).

3. Fig 1 showing risk interval as “Dark shaded area” which conflicts with the description of this figure in line no 392-394.

Thank you very much for pointing out this. We have fixed this mistake. (page 21, notes to figure 1)

4. This study included 30 episodes of previous AMI hospitalization during year prior to start of study window (Table 1). Excluding old MI episodes from the study could provide more insight.

Because this makes up only 8% of the study episode of 391, we did not exclude it so as to make our results more comparable to those by Kwong et al whose 364 study episodes included 24% of patients with prior AMI hospitalization. Nevertheless, your point is sound, and we did a stratified analysis. We found that, excluding those with prior MI episodes, the incidence rate ratio was 9.36 (6.44-13.60). In comparison, those without prior AMI hospitalization had a much lower IRR of 3.56 (0.49-26.15). Although the p-value was 0.35 for the interaction term, we believe it could still suggest that timely medical management might have brought down AMI risk for those with prior events. Our findings match those of Kwong et al as they conducted the same stratified analysis. (page 16, lines 385 to 396)

5. Out of 391 influenza tests, we see a total 288 test by test type in Table 1. Where is other tests?

Unfortunately, not all test types are recorded. This part of the data came from free text with a significant amount of missing information. We mentioned this now as a limitation. (page 18, lines 439-440)

6. Among those tests, one is antibody test. Which antibody? Is it reliable to indicate an acute influenza infection?

Again, this was from free text and only showed the virus (Flu B). We agree that in and of itself this might not be an acute infection, but since it was only one case, and we were working with limited information, we decided to keep it.

7. Throughout the analysis, authors compared rural (115) with non-rural patients. It would be great if authors define rural in their description of the study design.

Yes. That is a wonderful suggestion. We have incorporated this into the method section. (page 6, lines 154-156)

8. As the 96% episodes included in this study are “male’, gender is not a strong factor to include in the comparison (line 234, 306 and table 4).

Thank you very much for pointing this out. We agree that with 96% of episodes included in the study occurred for male patients, our study does not have enough data to study the impact of gender. We included it mostly so that readers can see this limitation and are aware of it when comparing our findings to others. We further addressed this in our limitation section now to emphasize the lack of generalizability. (page 18, lines 440-442)

9. Overall, it would be an added value if authors clearly describe what new knowledge were added by this manuscript.

This is an excellent point. We had some summary in the abstract page previously. Now we have incorporated them into the conclusions section. (page 19, lines 462 to 465)

**Reviewer #2**: 1. Please choose one heading for the introduction section in line 77, either introduction or background.

We have corrected this. (page 3, line 70)

2. As this study is also exploring the mediating role of WBC and PC, I would suggest the inclusion of previous knowledge on WBC and PC count in relation to influenza and acute myocardial infarction in the introduction section and also in the discussion section.

Publications have concentrated on the relation between either WBC/PC and myocardial infarction or between influenza infection and changes in WBC/PC. Only a few case studies from the CDC have presented all three together. We agree with your excellent suggestion and have now discussed the mediating role of WBC/PC in the introduction. (page 4, lines 95-100)

3. Line 184-185: Is there any justification why age 75 years was taken as a cut-off point to dichotomize the age variable? Why did not the authors consider age as a continuous variable?

We should have made it clearer. Please see our justification on page 8, lines 195-196. Taking your suggestion, we now also have analyzed age as a continuous variable (page 15, lines 378-380). This has been added to the discussion section. (page 15, lines 366-380)

4. The aim of mediation analysis using a survival model (Table 4) is to adjust for potential variables. However, I would recommend elaborating the description of table 4 in the result section - what significant variables (including comorbidities) imply.

We agree with your recommendation and have included these in the result section. (page 13, line 322 to page 14, line 335)

5. As this study has tried to examine the impact of age, please discuss what other studies have found in the discussion section as well.

We concur. This has been added to the discussion section (page 15, line 368-373).

6. Line 311 – 312: Please include the p-value here to make it clearer that there was no significant difference between the IR between those having vaccination record and without a vaccination record.

Although we included the p-value of 0.61 in line 267 on page 11, we agree it would be better to presented again to improve clarity. (page 15, lines 358-359)

7. Line 333: 8.89 in model 1, not model 4. Please correct it.

How embarrassing. We apologized for the glaring carelessness and have corrected it. (page 17, line 412)

8. In conclusion, line 373-374, the risk to the patient of older age might be misleading as no statistical significance was observed.

We agree. We have removed it from the conclusion section.
